# Lifting Hospital Electronic Health Record Data Treasures: Challenges and Opportunities

**DOI:** 10.2196/38557

**Published:** 2022-10-21

**Authors:** Alexander Maletzky, Carl Böck, Thomas Tschoellitsch, Theresa Roland, Helga Ludwig, Stefan Thumfart, Michael Giretzlehner, Sepp Hochreiter, Jens Meier

**Affiliations:** 1 Research Department Medical Informatics RISC Software GmbH Hagenberg Austria; 2 JKU LIT SAL eSPML Lab, Institute of Signal Processing Johannes Kepler University Linz Austria; 3 Department of Anesthesiology and Critical Care Medicine Kepler University Hospital GmbH Johannes Kepler University Linz Austria; 4 ELLIS Unit Linz, LIT AI Lab Institute for Machine Learning Johannes Kepler University Linz Austria

**Keywords:** electronic health record, medical data preparation, machine learning, retrospective data analysis

## Abstract

Electronic health records (EHRs) have been successfully used in data science and machine learning projects. However, most of these data are collected for clinical use rather than for retrospective analysis. This means that researchers typically face many different issues when attempting to access and prepare the data for secondary use. We aimed to investigate how raw EHRs can be accessed and prepared in retrospective data science projects in a disciplined, effective, and efficient way. We report our experience and findings from a large-scale data science project analyzing routinely acquired retrospective data from the Kepler University Hospital in Linz, Austria. The project involved data collection from more than 150,000 patients over a period of 10 years. It included diverse data modalities, such as static demographic data, irregularly acquired laboratory test results, regularly sampled vital signs, and high-frequency physiological waveform signals. Raw medical data can be corrupted in many unexpected ways that demand thorough manual inspection and highly individualized data cleaning solutions. We present a general data preparation workflow, which was shaped in the course of our project and consists of the following 7 steps: obtain a rough overview of the available EHR data, define clinically meaningful labels for supervised learning, extract relevant data from the hospital’s data warehouses, match data extracted from different sources, deidentify them, detect errors and inconsistencies therein through a careful exploratory analysis, and implement a suitable data processing pipeline in actual code. Only few of the data preparation issues encountered in our project were addressed by generic medical data preprocessing tools that have been proposed recently. Instead, highly individualized solutions for the specific data used in one’s own research seem inevitable. We believe that the proposed workflow can serve as a guidance for practitioners, helping them to identify and address potential problems early and avoid some common pitfalls.

## Introduction

**Figure 1:**
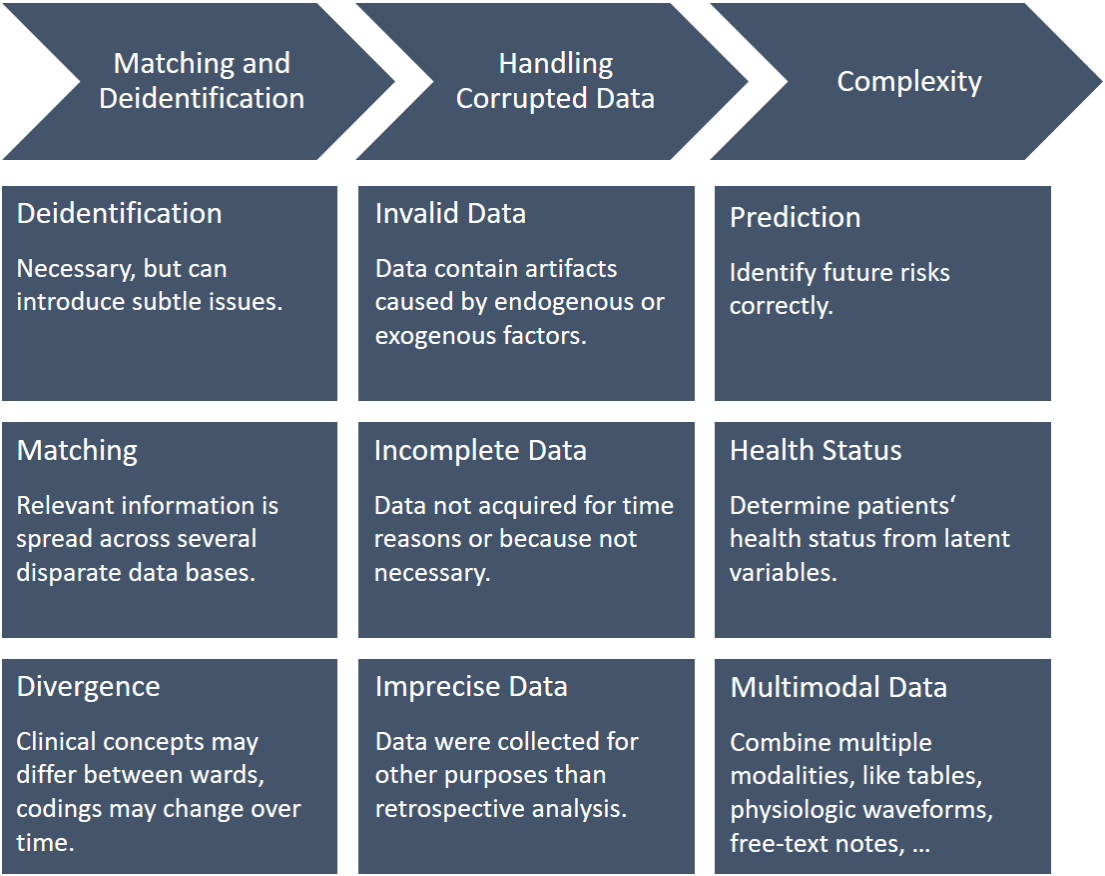
Primary challenges with retrospective medical data analysis (adapted from Johnson et al [18], which is published under Creative Commons Attribution 4.0 International License CC-BY 4.0 [19]).

Electronic health records (EHRs) contain a vast amount of information about an individual’s health status, including demographics, diagnoses, medication prescriptions, laboratory test results, high-frequency physiologic waveform signals, and others. Many prior studies have demonstrated how data science and machine learning (ML) can be applied to large databases of EHRs to successfully train models to predict many different patient-related outcomes, including mortality risk [1-4], length of hospital or intensive care unit (ICU) stays [1-3], cardiovascular decompensation [3,5,6], postoperative complications [7], and, recently, COVID-19 diagnosis and pathogenesis [8-12]. Although data preparation requires considerable time and effort [13,14], it is seldom represented in research outputs. One possible explanation could be that it is considered a “standard” task that always proceeds more or less the same and that can be automated to a large extent, thanks to readily applicable general purpose software tools [15-17]. In this paper, we illustrate through specific examples from a large-scale research project that this is not the case. Conducting a secondary (ML-based) analysis of raw EHRs from a hospital’s data warehouse is challenging in many respects due to several reasons. Above all, data were originally collected without any specific use case besides clinical application, and relevant information is usually distributed over multiple disparate databases that often lack comprehensible documentation. If clinical concepts (variables, categorical values, units of measurement, etc) are represented differently across distinct sources or if the coding of clinical concepts changes over time, data harmonization can become a real issue. Moreover, incomplete or invalid data, although a well-known problem in principle, can occur in many (unexpected) forms and might only be noticed after careful manual inspection. Figure 1 summarizes the main challenges with EHR data that we encountered in our work and are ubiquitous in retrospective medical data analysis [18].

Unlike many other papers about data preparation in a medical context, this work does not propose a novel generic data processing tool. Instead, we report the challenges that we faced and the lessons we have learned in a recent large-scale data science project. We present specific examples of messy and corrupted raw data to create awareness that (medical) data preparation is a nontrivial, labor-intensive endeavor, despite an ever-growing set of generic tools. Finally, we present a general data preparation workflow for similar research projects to help practitioners avoid the most common pitfalls.

The literature on medical data preparation for large-scale secondary (ML-based) analysis is scarce. Most studies have focused on model development and the final predictive performance of the developed models and only mention a few fundamental aspects of the data preparation pipeline. This is particularly true for the work of Rajkomar et al [1], but for a good reason: deep neural networks are used to learn “good” representations of the data in an end-to-end fashion, relying on the networks’ ability to automatically handle messy data properly. The pipeline is based on Fast Healthcare Interoperability Resources [20], meaning that all data available in this format can be readily processed without further ado—no feature selection, harmonization, or cleaning is necessary. Although appealing at first glance, the proposed approach has some limitations, as noted by the authors. Most importantly, deep neural networks often require massive amounts of data and computing resources to learn good representations. Second, the lack of data harmonization potentially impairs transferability across research locations; for example, for validation. Moreover, to train models in a supervised manner, one must provide labels, and depending on the use case, these labels may be difficult to extract, reintroducing the need for data preparation. It is also unclear whether the models developed in the aforementioned study [1] would have performed even better, had the data undergone more thorough manual inspection and curation.

Other studies have proposed generic data processing pipelines that can be applied off-the-shelf to well-known ICU benchmark databases such as Medical Information Mart for Intensive Care (MIMIC) [21-23] and the telehealth ICU collaborative research database (eICU-CRD) [24]. The most prominent examples being MIMIC-Extract [15], FIDDLE [16], cleaning and organization pipeline for EHR computational and analytic tasks [17], and Clairvoyance [25]. The authors of FIDDLE and Clairvoyance claim that their systems are sufficiently general to accommodate not only data extracted from MIMIC-III and eICU-CRD but also any EHR data available in a particular form. This may be true to a large extent, but we experienced that cleaning messy, raw data and bringing them into the required standardized form is at least as labor intensive (in terms of implementation effort) as the subsequent “generic” preprocessing steps that FIDDLE and Clairvoyance cover. Sculley et al [14] termed this phenomenon *glue code antipattern*. In general, MIMIC and eICU-CRD may be excellent benchmark databases, but we found that “real-world” data exported directly from a hospital’s IT infrastructure pose many challenges that are not present in these databases.

Shi et al [26] presented a medical data cleaning pipeline that explicitly addresses some of the issues that we also encountered in our research. They considered laboratory tests and similar measurements and proposed manually curated validation rules for numerical variables and an automatic strategy for harmonizing (misspelled) units of measurement through fuzzy search and variable-dependent conversion rules. The focus of Shi et al [26] is on improving the quality of data [27-29], whereas Wang et al [15], Tang et al [16], and Mandyam et al [17] are mainly concerned with transforming data into a form suitable for ML. A more detailed evaluation of FIDDLE, MIMIC-Extract, and cleaning and organization pipeline for EHR computational and analytical tasks and the approach to our data by Shi et al [26] can be found in Multimedia Appendix 1 [15-17,26].

The extensive survey article by Johnson et al [18] summarizes the main issues of medical data analysis similar to that in this work. The authors also established a high-level categorization of these issues into *compartmentalization*, *corruption*, and *complexity* (Figure 1) and argued that data acquisition and preparation in the critical care context are particularly difficult because data are collected for a different purpose.

Sendak et al [30] arrived at similar conclusions, noting in particular that solutions developed for one site did not scale well across multiple sites because of redundant data validation and normalization. The authors provided estimates for the expected cost of deploying a model to screen patients with chronic kidney disease in other hospitals. We refrain from extrapolating such estimates from our findings but agree that the costs for preprocessing data from other sites into a form suitable for existing prediction models will likely be significant.

## Methods

### Data Preparation

Raw EHRs stored in hospitals’ data warehouses cannot readily be used for developing clinical prediction models but must first be extracted, analyzed, and subjected to a series of preprocessing steps. These steps may differ between data modalities and sources but usually include some sort of *validation* (ensuring data accurately reflect reality), *harmonization* (establishing uniform representation of equivalent concepts), and *transformation* (bringing data into a form suitable for model development, eg, extracting useful information). Furthermore, it must be ensured that a sufficient number of data points are available in the first place and that clinically meaningful target labels can be extracted from them in the case of supervised learning. We demonstrate how this can be accomplished in a disciplined, effective, and efficient manner by referring to a specific data science project.

### Underlying Data Science Project

All results presented in this paper originate from a large-scale data science project for developing data-driven clinical prediction models. Specifically, the following 5 use cases were considered: (1) optimizing patient throughput in the ICU, (2) increasing the accuracy of treatment priorities in emergency medicine, (3) improving the selection of blood products, (4) predicting patient deterioration in the ICU to enable preventive interventions, and (5) predicting COVID-19 infections using routinely acquired laboratory tests [11]. All use cases were based on retrospective, routinely collected data from the Kepler University Hospital, a large university hospital in Linz, Austria. A wide variety of data modalities were used, including patient demographics, laboratory tests, diagnoses, vital signs, and even high-frequency physiological waveform signals. Information represented by natural-language text was mostly ignored (except for short free-text diagnoses), and imaging modalities were excluded altogether.

The amount of data varies among the 5 use cases; for instance, use cases 1 and 4 are naturally confined to patients admitted to the ICU, whereas for use case 2, only patients who visited the emergency department (ED) could be taken into account. The period covered by the data also depends on the use case. Table 1 lists the particular period and the total number of patients for each of the 5 use cases. Altogether, the order of magnitude of the number of data items processed was 10^9^ (excluding high-frequency waveform data) of which vital signs and laboratory tests constituted the vast majority.

The specific results of the 5 use cases were not the main focus of this paper. Instead, the use cases serve merely as illustrative examples throughout the remainder of this paper.

**Table 1:** Use cases considered in the research project^a^.

Use case	Short description	Period	Patients, n
1	Optimizing ICU^b^ patient throughput	2010-2020	14,236
2	Increasing the accuracy of treatment priorities in ED^c^	2015-2020	77,972
3	Improving the selection of blood products	2016-2020	5855
4	Predicting patient deterioration in the ICU	2018-2020	3069
5	Predicting COVID-19 infections [11]	2019-2020	79,884

^a^Note that patient cohorts partly overlap.

^b^ICU: intensive care unit.

^c^ED: emergency department.

### Data Sources

Relevant data for the 5 mentioned use cases are contained in 3 central data management systems in the hospital’s IT infrastructure: the *hospital information system* (HIS), *patient data management system* (PDMS), and *Bedmaster system*. The HIS is a hospital-wide data warehouse that contains information on all patients admitted to the hospital. Among others, this includes demographics (date of birth, sex, etc), detailed information about in-hospital transfers, diagnoses, laboratory test results, and intramural mortality. PDMS is deployed in 5 ICUs associated with critical care in the hospital. Hence, it only contains information about patients admitted to the ICU during their hospital stay but complements the basic information found in the HIS with automatically recorded vital sign measurements (heart rate, blood pressure, body temperature, etc; up to 30 measurements per vital sign per hour), precise information about administered medications, and manually recorded scores (eg, Glasgow Coma Scale). The Bedmaster system [31] can be connected to bedside monitoring devices and automatically stores the vital signs, physiological waveforms, and alarms produced by these devices. The temporal resolution of the acquired data far exceeds the resolution in PDMS, with vitals being recorded every 2 seconds and waveforms sampled at rates of 60 to 240 Hz. This system is only deployed in 2 of the 5 ICUs and was installed in March 2018. Hence, the number of patients covered by it is significantly smaller compared with HIS and PDMS.

**Figure 2:**
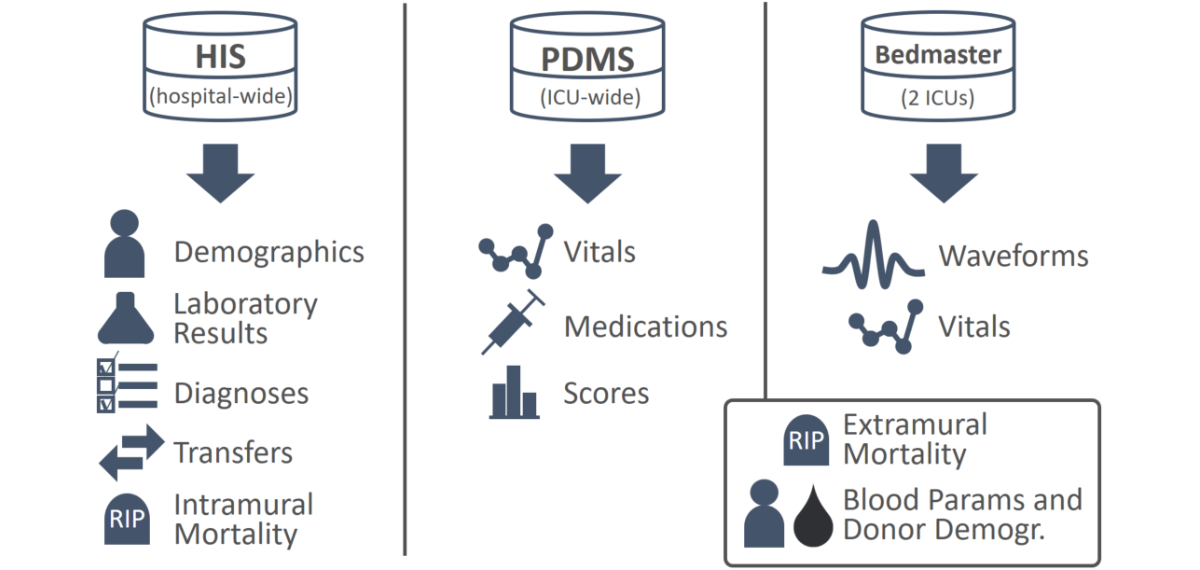
Data sources and exported modalities in use cases 1 to 5. HIS, PDMS, and Bedmaster are data management systems deployed in the hospital, whereas information about extramural mortality and blood products had to be obtained from external sources. HIS: hospital information system; PDMS: patient data management system; ICU: intensive care unit.

In addition, information about the extramural mortality of patients after hospital discharge was obtained from the Austrian Federal Statistics Agency (use case 1), and information about blood products transfused in the hospital was obtained from a local blood bank (use case 3). Figure 2 summarizes all data sources and modalities used in the 5 cases.

### Ethics Approval

For each use case mentioned in this work, approval was obtained from the Ethics Committee of the Medical Faculty, Johannes Kepler University, Linz, Austria. The corresponding study numbers are 1015/2021, 1233/2020, 1232/2020, 1014/2021, and 1104/2020.

## Results

### Data Overview

Before beginning to export raw data from their hospital-internal storage, it is imperative to obtain an overview of what kind and how much data are available. This might sound obvious but can be more intricate than it seems. For example, the number of patients or cases at one’s disposal is not always indicative of the amount of suitable data. Specifically, in the common setting of supervised learning, only data points to which clinically meaningful target labels can be assigned are useful. In the blood transfusion use case 3, for instance, the types of transfusion-related complications we could consider were limited by the availability of sufficient pre- and posttransfusion laboratory measurements to identify the respective complications. Ultimately, sufficient labeled training samples could only be generated for predicting acute kidney injury and acute respiratory failure. Other organ systems, although interesting in principle, had to be excluded from the analysis. Acute respiratory failure also had to be excluded eventually because the class imbalance was found to be too strong.

Given the richness of information stored in EHRs, there are normally enough data that can be converted into features that clinical prediction models may attend to. However, one must be aware that information accumulates over time, meaning that more data about a patient are available toward the end of the hospital or ICU stay than toward the beginning. For us, this was especially relevant in use case 2, where treatment priorities and 30-day mortality of patients visiting the ED had to be predicted based on only a few pieces of information typically recorded in the ED.

### Defining Labels for Supervised Learning

Routinely collected retrospective EHR data do not always contain information about the outcomes that one wants to predict. Typical outcome parameters, apart from mortality or length of hospital stay, are often composites of several parameters that must be deduced from surrogate variables. Some authors, for instance, resort to hypotension as an indicator of cardiac instability [5,6], an approach we adopted in our use case 4 for predicting patient deterioration. Similarly, widely accepted criteria for organ system failure exist; for example, Kidney Disease: Improving Global Outcomes [32] for kidney disease and the Berlin definition for acute respiratory distress syndrome [33]. Both were used in use case 3.

Further problems can arise when trying to predict the effects of interventions. First, it might not always be possible to connect an observed outcome to a specific intervention, especially if multiple interventions occur within a short time. In the blood transfusion use case 3, in many cases, 2 or more blood products are transferred simultaneously, rendering it impossible to determine which of the administered transfusions are responsible for a posttransfusion complication. In such a situation, framing the prediction task as a *multiple instance learning problem* [34] might be the only remedy. Second, if the goal is to assess or improve existing clinical decision policies, one is confronted with questions such as “What would have happened to the patient if he/she had been treated differently?” Naturally, such questions are difficult to answer based on retrospective data in which interventions and treatments are fixed, and counterfactual trajectories cannot be explored, although the literature on estimating counterfactual treatment outcomes through statistical analysis and ML exists [35]. In use case 1, where the primary goal was to predict the optimal time for discharging ICU patients back to a ward, we resorted to answering the proxy question of whether transferred patients should have better stayed longer in the ICU. We determined this by identifying patients who died or returned unexpectedly shortly after ICU discharge.

### Accessing and Extracting the Data

Hospital IT infrastructure is usually designed to provide easy access to the data of individual patients to deliver optimal care. Unfortunately, this does not imply that batches of data from distinct patients can be accessed, let alone extracted, easily. In particular, if the amount of manual interaction required for exporting data is too high, individual retrospective studies might be feasible, but the automatic real-time deployment of prediction models on live data may not be feasible. Data access can be challenging when there is only one source but even more so if there are multiple disparate data sources one must incorporate. In our project, we had to access 3 distinct databases: HIS, PDMS, and Bedmaster (Figure 2). HIS is a SAP-based system from which tables can be exported as CSV or Microsoft Excel files, and PDMS is a PostgreSQL relational database that allows exporting the results of queries in whatever table format is desired. In contrast, exporting data from the Bedmaster system turned out to be cumbersome because only XML and JSON exports are supported by default. Representing the massive amount of waveform and vital sign data in either of these verbose formats resulted in huge files that could not be processed efficiently; so, in the first step, we had to extract the relevant numerical values from the JSON files and store them in the more efficient HDF5 format. This process was considerably more intricate than anticipated because of inconsistencies in the exported data representation that are detailed in Multimedia Appendix 2 [36-39].

### Matching Data From Different Sources

Data exported from different sources must be matched to obtain coherent records of the patients or cases under consideration. Under normal circumstances, this is straightforward because of common identifiers. However, according to our experience, such identifiers do not always need to be present or change over time. Specifically, data exported from the Bedmaster system lack identifiers, such as patient or case IDs. Knowing only the ICU bed they stem from, as well as the precise timestamp of each single recorded value, we had to assign the corresponding IDs manually based on the information about which patient occupied which ICU bed at which time. This approach works but is cumbersome and adds extra complexity and is another potential source of mistakes. It is also more difficult to automate than simply joining tables on common ID columns.

Mappings between identifiers and the entities they refer to may change over time as experienced in our project with drug codes. Every drug has a unique code that is used to reference it in prescription tables, but for unknown reasons, the coding changes at certain points in time. The precise information when this happens is stored in another table, so that drug names *can* be recovered from the provided codes and timestamps of prescriptions. Yet again, the whole process is not as straightforward as we would have hoped.

### Deidentification

Sensible personal information stored in EHRs can only be shared in a deidentified form. There are no universal rules *on how* data need to be deidentified, as long as identifying individual patients afterward becomes practically impossible. In our project, deidentification amounted to removing patient names and replacing hospital identifiers, such as patient IDs or case IDs, with project-internal identifiers that could be used to match corresponding data items across different tables. Furthermore, all timestamps were shifted by a random per-patient offset in the future to avoid reidentification of patients from knowing their exact admission or discharge times. Timestamps were shifted after matching data from different sources because some matching strategies depend on precise temporal information, as described earlier. The timestamps were shifted such that the time of day and day of week were preserved because both constitute potentially valuable information for downstream data analysis tasks. The same is true for seasonality, which was also roughly preserved. This deidentification policy is analogous to that used for MIMIC-III [21]. We remark that it is not as thorough as the policy implemented for releasing the more recent AmsterdamUMCdb [40]: there, theoretical concepts such as *k*-anonymity and *l*-diversity are considered to render reidentifying individual patients practically impossible under advanced threat models assuming “rogue researchers” and “rogue insurance companies” with access to the data. As, in our case, all data (even in deidentified form) are kept private and can only be accessed by project members, we did not deem such a thorough deidentification policy necessary.

Deidentification removes or replaces information that can otherwise be used to detect inconsistencies in the data, such as the same patient ID being accidentally assigned to multiple patients with different names. Therefore, it is crucial to ensure that any problems of this kind are detected and corrected either before or while deidentifying the data when the necessary information is still available. Specifically, we implemented extensive sanity checks that, for instance, ensure case and patient IDs are in a 1:n relationship (every case ID corresponds to a unique patient ID, but a patient ID can have multiple case IDs associated with it). All instances violating this principle are immediately reported to the human operator, allowing him or her to either overwrite one of the identifiers or discard the instances completely. Furthermore, missing patient IDs were automatically reconstructed from known case IDs whenever possible. The availability of patient IDs is essential because the random temporal offsets used for deidentifying timestamps are associated with patient IDs rather than case IDs. Finally, because hospital-assigned case IDs follow a clearly defined pattern that allows them to be distinguished from patient IDs, accidentally swapped case IDs and patient IDs are automatically exchanged before deidentification.

The kind of information that should be preserved by deidentification depends very much on the prediction task one wants to tackle. For example, in our approach, the temporal order of the data is preserved only within a patient but not across all patients. In particular, the total number of patients in the ICU at a certain point in time, a potentially relevant input for use case 1, can no longer be determined after deidentification. For the same reason, it is impossible to detect *domain shifts* in the deidentified data, which are systematic changes in the distribution of the data over time (domain shifts can be caused by many different factors such as new measurement equipment, laboratory test procedures, or changes in the prevalence of diseases in the patient population). Therefore, all relevant temporal features that could not be computed after deidentification had to be extracted and added to the data before deidentification.

### Inspection and Exploratory Analysis

Real-world data can be corrupted or otherwise ill-behaved in many unforeseeable ways, in addition to well-known issues related to missing values or invalid measurements, that a thorough inspection and exploratory analysis is inevitable. Indeed, in our experience, this is one of the most labor-intensive tasks in the entire data preparation pipeline. Owing to the nature of the problem, it is difficult to devise general rules for what one should pay attention to. Instead, we report one particularly subtle issue encountered in our work. It might be specific to our hospital but is meant to serve as an illustrative example of what can unexpectedly happen when working with EHRs. More examples can be found in Multimedia Appendix 2.

**Figure 3:**
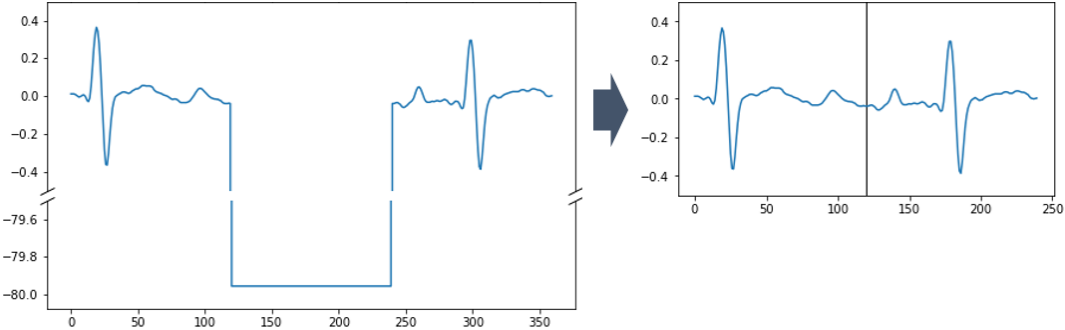
Short periods of constant low values in waveform signals might have to be cut out. Left: original signal with a 0.5-second period of constant low values. Right: signal after cutting out the low value; as can be seen, the 2 ends of the signal fit perfectly.

In use case 4, we made heavy use of physiological waveform signals, such as electrocardiogram, arterial pressure, and oxygen saturation, to predict whether the condition of ICU patients will deteriorate within the next 15 minutes. Waveform signals are recorded by the Bedmaster system and can be exported as arrays of numerical values. It should be clear that because of the way in which these data are measured, there can be many types of measurement artifacts in the signals; that is, highly unusual waveform morphology caused by slipped sensors, or patient movements. This must be expected and addressed either explicitly by automatically detecting periods of invalid waveform data [36] or implicitly by relying on the subsequent ML algorithm’s ability to learn how to differentiate between normal and abnormal signals. An entirely unexpected issue is depicted in Figure 3: occasionally, the signals assume constant low values for a short time. The natural guess of measurement errors (eg, caused by slipped sensors) is likely wrong because simply cutting out the constant low-value period leads to smooth curves in all inspected cases. Such situations may thus indicate data artifacts of unknown origin that must be removed to obtain coherent signals, but strangely they do not always occur in all simultaneously recorded waveforms at the same time. Therefore, we opted to refrain from cutting out fragments of the raw signals to avoid a possible temporal misalignment of different waveforms.

### Implementation

Eventually, the pipeline for extracting and preprocessing all relevant EHR data must be implemented in the actual code. This can be challenging in many respects. First, it is tempting to make extensive use of technologies aimed at rapid prototyping (eg, Jupyter notebooks) to quickly experiment with the data and preprocess them for a particular use case in a particular hard-coded way. This might work well in the short term; however, in the long term, a structured modular codebase that allows the exchange of individual components and adjustment (and logging!) of configuration settings is the better approach. In particular, the logging of configuration settings is of utmost importance to know exactly how data were preprocessed and how models were generated, thereby obtaining reproducible results.

Second, pipelines implemented to process a specific data modality for a particular use case should be reusable in other use cases that depend on the same data modality, at least to a certain extent. Even if the desired output format of the data after preprocessing differs between the 2 use cases, there are almost certainly some steps in the pipeline that are applicable to both. Reusing existing functionality rather than reimplementing it enables a consistent treatment of data across use cases and as a side effect may even help to abstract from the peculiarities of one use case and implement preprocessing functionality in a more general way. For example, we used laboratory test results in each of the 5 use cases either as features or for assigning labels (or both). In use case 4, the last 3 measurements of a fixed set of laboratory parameters relative to a given point in time are used as features, whereas in use case 3, the last measured value of a certain parameter before a blood transfusion is compared with measurements after the transfusion to determine whether it incurred a complication. Both are special cases of the more general principle of finding the last or first *n* measured values before or after a given point in time and could hence be implemented in one common function.

Finally, the inclusion of general-purpose third-party tools in the data preparation pipeline clearly has its benefits as well as potential downsides. On the one hand, functionality implemented therein does not have to be reimplemented (nor tested) from scratch, but on the other hand, Sculley et al [14] point out that it may lead to many glue code and pipeline jungles for bringing data into the right shape. In our project, we restricted ourselves to well-established libraries from the Python ecosystem, including NumPy [41], Pandas [42], and scikit-learn [43] and deliberately avoided tools such as FIDDLE [16]. The former 3 are libraries of useful classes and functions that can be easily integrated into one’s own pipeline. The latter implements a full medical data preparation pipeline itself, which, although being generic and customizable in principle, did not offer the amount of flexibility we would have required to accommodate our data.

More precisely, our data preparation pipeline consists of 3 main steps: harmonization, validation, and transformation. Harmonization, that is, ensuring that equivalent concepts are represented consistently, is very specific to each data modality and typically amounts to assigning unique names to equivalent variables and converting measured values into a common unit of measurement. Validation of recorded values happens with respect to manually specified, threshold-based rules. Analogous to Harutyunyan et al [3], we distinguished between invalid numerical values and extreme outliers. Each validation rule is characterized by 2 ranges *r1⊆r2*, where everything inside *r1* is deemed admissible and everything outside *r2* is deemed an extreme outlier. Extreme outliers *x*∉*r*2 are deleted entirely, whereas the values *x*∈*r2*\*r1* are set to the nearest admissible value in *r1*. Finally, transformation also depends on the specific data modality under consideration but is often concerned with resampling EHR tables in an event-based entity-attribute-value format into a more ML-friendly wide table format. This proceeds by aggregating all observations within a given time window with respect to a fixed set of rules, such as taking the mean, sum, or temporally last of all the measured values. If a variable has not been measured at all in a time window, the “missing” recordings are imputed. As other authors have noted [44], clinical measurements are not missing at random; therefore, explicit *missingness masks* indicating whether a value has been imputed are added as extra features. In general, one must also be careful when imputing the mean or median of all observed values, as this could introduce bias. For example, if a variable is only measured if a patient has a certain condition, the measured values are not representative of the entire population.

## Discussion

### Principal Findings

The preceding sections illustrate that the preparation of EHRs for secondary analysis and the development of prediction models constitute a challenging endeavor. In addition to the well-known ubiquitous data problems for which generic off-the-shelf solutions exist (eg, imputation of missing values), we identified many issues in our raw data that had to be addressed individually. Even worse, none of these issues could be expected or popped up during the first quick scan of the data but instead were discovered only after a thorough exploratory analysis. Different kinds of patient identifiers being accidentally swapped is certainly something one would not expect at all, yet we found a few such cases in our data. The use of multiple codes or names for the same clinical concept is also not trivial to detect, especially if it is a mere artifact of the internal data representation that does not surface in clinical practice. If the mapping between codes and concepts changes over time, data harmonization becomes a true challenge. With regard to data validation, blindly discarding all nonnumeric values of a supposedly numeric variable fails to account for censored values such as “>120.0” (Multimedia Appendix 2) that do carry useful information. Finally, the subtle issues with waveform data reported above not only demand a thorough systematic analysis of timestamps and measured values but are also difficult to fix. Altogether, these observations support our claim that although generic tools such as FIDDLE [16] and Clairvoyance [25] doubtlessly do have their merits, one must be careful not to underestimate the additional effort of modality- and source-specific data analysis and preparation. In general, we believe that extensive libraries of well-documented, generic, and cleanly implemented functionalities focusing on the peculiarities of medical data preparation (harmonizing and validating physiological variables, resampling event-based entity-attribute-value tables into wide tables, etc) are more valuable than full-fledged end-to-end pipelines, regardless of how generic and configurable they are.

Extracting labels that indicate the outcome of interest from retrospective data can be more intricate than one might expect. Often, these outcomes (patient deterioration, organ system failure, optimal treatment policy, etc) are not explicitly recorded in EHRs and must therefore be approximated. The quality of such an approximation might influence not only the performance of the generated prediction models but also their applicability to clinical practice. Furthermore, if the definition of some label depends on scarcely recorded variables, only a few labeled samples may remain. In such a situation, methods based on *self-supervised and semisupervised learning* [45-47] might be the only remedy.

EHRs contain highly sensitive patient information that, for good reasons, must be deidentified before it can be shared with scientific partners in research projects. How and to what extent this needs to be carried out often not clearly defined, especially regarding the treatment of temporal information. Temporal data may contain highly relevant information depending on the concrete use case. On the one hand, knowing the time of day and day of week of a particular event is necessary if the prediction task at hand has to take clinical routines into account; on the other hand, knowing the (rough) order of events across different patients enables detecting domain shifts in the underlying data distribution. Finally, if the use of a particular resource at any given point in time is of interest, this information must be extracted before deidentifying the timestamps, or timestamp deidentification must be avoided entirely. In our experience, it is good to first determine the kind of information one needs for a particular use case and then devise deidentification strategies that preserve as much of the previously determined information as possible while observing legal regulations and hospital-internal restrictions.

Finally, if the ultimate goal of developing prediction models is to deploy them in clinical practice, data access becomes a factor that must be considered. The more manual steps involved in exporting the data from the hospital IT infrastructure into the desired format, the more difficult real-time deployment will be. In our use case 4, automatically exporting the necessary data of all current ICU patients after every *n* minutes and then promptly processing them is challenging and currently work in progress. This mainly owes to the fact that the entire data warehousing system of the Kepler University Hospital was designed for clinical use rather than real-time analysis. However, alternatives exist; a sophisticated solution for efficient storage of and access to medical data for data science projects is presented in a study by McPadden et al [48].

### Workflow

**Figure 4:**
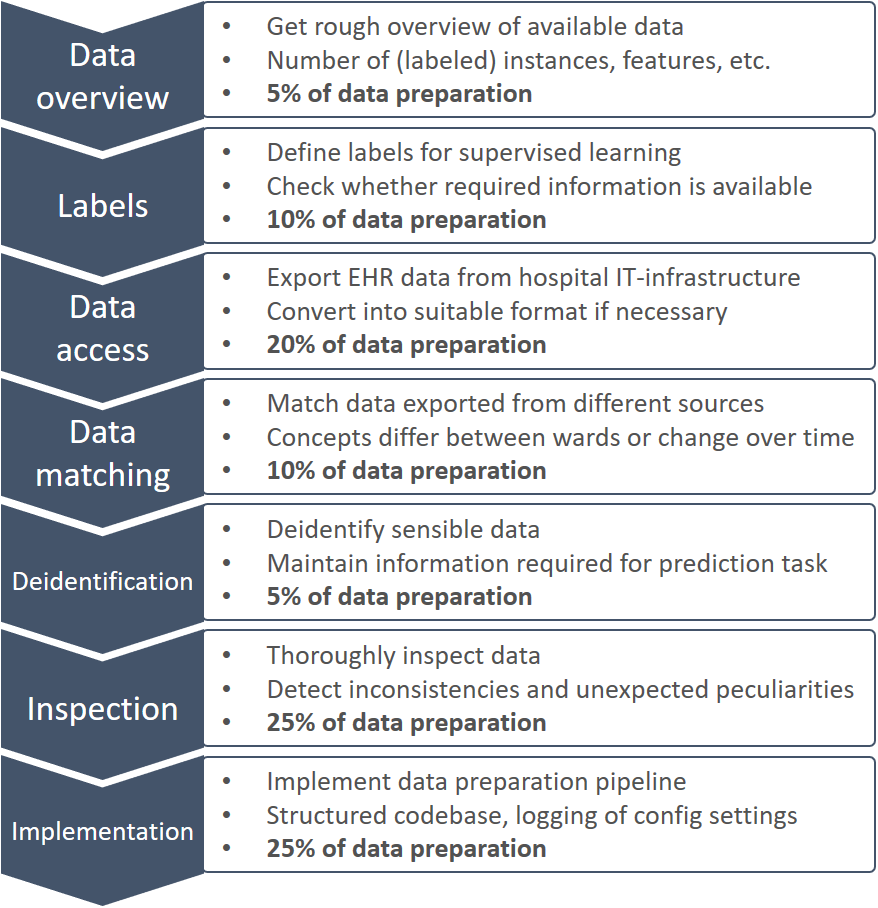
Data preparation workflow for retrospective EHR data analysis. EHR: electronic health record.

The data preparation workflow we followed in our project is summarized in Figure 4, with rough estimates of the relative time and effort taken by the individual steps. We think that it generalizes to other data science projects with retrospective EHR data and hope that it can serve as guidance for other researchers to identify and address potential problems early and avoid some common pitfalls.

The presentation of the (linear) workflow in Figure 4 is simplified because in reality, there are many feedback loops. For instance, inspecting the data may reveal issues that can only be rectified if additional information is extracted from the system, and some issues might only surface after developing the first prediction models.

It is important to note that the results presented in this paper only refer to data preparation for subsequent model development but not to the development and validation of actual prediction models. We think these are “standard” tasks in data science and ML that are not specific to medical data. However, we do acknowledge that selecting the appropriate class of prediction models for a given task, optimizing hyperparameters, and training models in the right way are by no means trivial and require a lot of time and effort. This is also true for deploying models in clinical practice, where topics such as *handling domain shifts*, *detecting out-of-distribution data*, and *explaining model decisions in a manner comprehensible to patients* must be addressed. Things become even more difficult if existing models are to be deployed in other hospitals because most of the steps in the above workflow must be repeated. Only the definition of labels and (possibly) deidentification can be skipped, and some parts of the existing pipeline implementation can perhaps be reused. According to our rough estimate, approximately 75% of the effort invested in the initial data preparation for developing prediction models must be reinvested for each hospital that these models are deployed. As noted in a study by Sendak et al [30], this incurs significant additional costs.

### Conclusions

Preparing raw medical data from productive environments for retrospective analysis and ML remains challenging and time consuming. Our findings suggest that real-world EHR data can be messy and corrupted in so many subtle ways that thorough exploratory analysis and tailor-made preprocessing functionality for the data at hand are inevitable. We want to create awareness of this fact and hope that the sketched data preparation workflow becomes a valuable guidance for future large-scale data science projects involving routinely acquired medical data.

## Appendix

Multimedia Appendix 1 Brief evaluation and comparison of four EHR data preparation tools.

Multimedia Appendix 2 Overview of the data modalities used in our research and specific issues encountered when processing them.

## Abbreviations

ED: emergency department
EHR: electronic health record
eICU-CRD: telehealth ICU collaborative research database
HIS: hospital information system
ICU: intensive care unit
MIMIC: Medical Information Mart for Intensive Care
ML: machine learning
PDMS: patient data management system
