# Supplementary material for: Lifting Hospital Electronic Health Record Data Treasures: Challenges and Opportunities
Source: JMIR Med Inform. 2022 Oct 21;10(10):e38557. doi: 10.2196/38557 (PMC9636533; doi:10.2196/38557)
Supplement: Multimedia Appendix 1 [file medinform_v10i10e38557_app1.docx]

# Multimedia Appendix 1

## Evaluation of Generic Data Preparation Tools

Supplementary Table 1 presents a detailed evaluation of four EHR data preparation tools, in terms of coverage of the specific problems and tasks we encountered in our work. One must keep in mind, though, that MIMIC-Extract and COP-E-CAT were developed specifically for MIMIC-III and MIMIC-IV, respectively, and so cannot easily be applied to other data. Furthermore, although MIMIC-Extract implements sample generation and label extraction for four standard use cases, it does not cover our particular use cases at all. For a fair comparison, one must note that each of these tools supports other aspects of data preparation that were not relevant to us, and are hence missing in the table. One example is cohort selection, which we took care of immediately when exporting the data.

Supplementary Table 1. Detailed evaluation of FIDDLE [16] (F), MIMIC-Extract [15] (M), COP-E-CAT [17] (C) and
Shi et al. [26] (S).

| Problem / Task | F | M | C | S |
| --- | --- | --- | --- | --- |
| ID-related issues |  |  |  |  |
| Multiple names/codes for same variable |  | ✓ | ✓ |  |
| Values of distinct variables are swapped |  |  |  |  |
| Mixture of numerical and categorical values | ✓ |  |  |  |
| Multiple (misspelled) UOMs for same variable |  | ✓ | ✓ | ✓ |
| Multiple codes for same categorical value |  |  |  |  |
| Prefixed numerical values (e.g., ‘>150.0’) |  |  |  |  |
| Mixture of static and dynamic variables | ~ |  |  |  |
| Mixture of isolated and continuous interventions |  |  |  |  |
| Onset and duration of effect of interventions |  |  |  |  |
| Group similar concepts | ~ | ~ |  |  |
| Coding of variables changes over time |  |  |  |  |
| Extract structured information from free-text notes |  |  |  |  |
| Waveform-related issues |  |  |  |  |
| Threshold-based validation |  | ✓ | ✓ | ✓ |
| Resample into regular time-grid | ✓ | ✓ | ✓ |  |
| Extract last *n* measurements |  |  |  |  |
| Extract binary indicators for interventions |  | ✓ | ✓ |  |
| Impute missing values | ✓ | ✓ | ✓ |  |
| Extract samples and labels for concrete use case |  |  |  |  |
