# Supplementary material for: Lifting Hospital Electronic Health Record Data Treasures: Challenges and Opportunities
Source: JMIR Med Inform. 2022 Oct 21;10(10):e38557. doi: 10.2196/38557 (PMC9636533; doi:10.2196/38557)
Supplement: Multimedia Appendix 2 [file medinform_v10i10e38557_app2.docx]

# Multimedia Appendix 2

## Data Modalities used in the Research Project

### Demographics

Information about patient demographics constitutes the most well-behaved data modality. It is stored in simple tables, where each row corresponds to a patient and each column to an attribute (date of birth, sex, etc.). The number and names of columns vary depending on the source database, since information that is more detailed is available for ICU patients. We handle such variations of the table format by renaming columns w. r. t. a manually defined mapping to obtain a fixed set of column names but do not automatically add columns missing in non-ICU tables.

In most cases, validation of patient demographics proceeds by discarding values exceeding certain hand-crafted thresholds. This approach in particular applies to height and weight, which must be both positive and less than some physiologically meaningful maximum values (250 cm and 400 kg, respectively, in our case). However, we found that in rare cases, height and weight are accidentally swapped, and so we implemented a simple mechanism that identifies all patients whose weight, in kilograms, exceeds their height in centimeters. Weight and height of these patients are then exchanged. Incorporating more sophisticated approaches to weight and height validation, as proposed by Phan et al. [37], is left for future work. In particular, our current approach does not account for morbid and super obese patients whose weight (in kilograms) might indeed exceed their height (in centimeters). We ruled this possibility out for the specific patient cohort we were working with.

### Laboratory Tests

Laboratory test results are exported in an event-based format, where every row in the table corresponds to a single measurement of some lab parameter at a particular time; see Supplementary Table 1 for an example. One obvious drawback of such a representation is the mixture of numerical and categorical values in the ‘value’ column. This makes it difficult to determine whether a specific lab parameter assumes numerical or categorical values and what the possible classes are in the latter case. Even worse, if a lab parameter assumes both numerical and categorical values, this might either indicate that the variable is categorical and numbers represent some classes (think of blood type, with classes A, B, AB, and 0) or that it is numerical and non-numerical values are invalid (like in row 3 of Supplementary Table 1, where the value of creatinine is ‘s. Komm.’ – which means ‘see comment’). Therefore, in an initial exploratory data analysis, we compiled lists of all numerical and categorical variables occurring in our lab data and determined the possible classes of categorical variables. We then hard-coded this information in the lab data preprocessing pipeline.

Supplementary Table 1. Example snippet of raw lab results. Every row corresponds to one measurement and is characterized by a case identifier, a timestamp, a short code describing the measured parameter, a measured value, and an optional unit of measurement. BGR: blood type; KREA, KREP: creatinine; AKST: antibody search test; TZ: thrombin time; uom: unit of measurement.

|  | case_id | timestamp | code | value | uom |
| --- | --- | --- | --- | --- | --- |
| 0 | 59036 | 2235-07-14 10:48:32 | BGR | A |  |
| 1 | 59036 | 2235-07-14 10:48:32 | KREA | 0.9 | mg/dl |
| 2 | 59036 | 2235-07-14 17:13:05 | KREP | 1.0 | mg/dL |
| 3 | 59036 | 2235-07-14 17:13:06 | KREA | s. Komm. | mg/dL |
| 4 | 11067 | 2178-10-02 08:24:58 | BGR | 0 |  |
| 5 | 11067 | 2178-10-02 08:24:42 | AKST | negative |  |
| 6 | 11067 | 2178-10-05 08:56:20 | AKST | neg. |  |
| 7 | 38354 | 2178-10-05 08:57:46 | TZ | 47.4 | s |
| 8 | 38354 | 2204-02-22 07:01:14 | TZ | >150.0 | s |

Another big issue with our lab data is that different codes can be used for the same concept, like for the codes of lab parameters and codes used for classes of categorical variables and units of measurement (UOMs). In Supplementary Table 1, rows 1 to 3 all correspond to measurements of serum creatinine, although the two different codes, KREA, and KREP, are used. Moreover, the UOM differs (lower-case ‘l’ vs. upper-case ‘L’), which might appear a trifle but is something that must be taken into account when deciding whether two syntactically distinct UOMs are equivalent or whether the corresponding measured values must be converted into a common UOM. Finally, the two values ‘negative’ and ‘neg.’ in rows 5 and 6 have the same meaning. Rows 7 and 8 of Supplementary Table 1 reveal yet another subtlety about measured lab values: prefixes, like ≤ and >. Note that in this case, the measured lab parameter TZ (thrombin time) is just an ordinary numerical variable, and >150.0 merely indicates that the measured value is not known precisely. During preprocessing, we extract prefixes of values of numerical variables and replace the corresponding numerical values by smaller or larger values selected based on medical expert knowledge. In other cases, however, prefixed values might indicate separate classes of categorical variables, again highlighting the need for a hard-coded list of (categorical) variables and their respective classes, as described above.

Values of lab parameters may change over time and are thus measured multiple times for a patient. Consequently, measurements lying in the distant past (say, more than a week ago) bear hardly any clinical relevance and should thus not be taken into account when training prediction models. Two notable exceptions of routinely acquired lab parameters that under normal circumstances remain constant over time are blood type and presence of the Rh(D) antigen (also known as *Rhesus factor*). Machine learning models may safely rely on measurements of these variables regardless of when they were made. We therefore treat blood type and Rhesus factor like static variables in our lab data preprocessing pipeline. In the rare cases where blood type and Rhesus factor may change, like after blood transfusions, we rely on them being measured again after the transfusion, because in any case the *last* available recordings are used.

### Medications

Raw medication data is similar to raw lab data because it is represented in an event-based format. However, the big difference between these two modalities is the ‘time-span’ nature of medications: drugs are usually administered intravenously over a certain period, possibly with dosage adjustments. An event, therefore, does not necessarily correspond to an isolated measurement at a particular time (as in lab data) but rather to a dosage change of a potentially still ongoing infusion. Supplementary Table 2 illustrates these considerations through a specific example. In particular, the first three rows refer to a single infusion, which lasted about 1.5 hours during which the dosage was adjusted once, shortly before the end. Column ‘dose’ contains the total dose of the drug administered since the last dosage change, and so is always 0 at the beginning of continuous infusions. The ‘end’ flag indicates the event in row 2 being the last event of this infusion, after which the dose is set to 0 until the next transfusion starts (if any). In contrast, row 3 corresponds to a bolus drug administration where the dose of the temporally first event is already set to the total given dose. This mixture of bolus and continuous drug administrations adds a fair amount of complexity that must be properly addressed during data preprocessing.

Supplementary Table 2. Example snippet of raw medication data. Every row corresponds to one dosage change and is characterized by a case identifier, an infusion identifier, a timestamp, a drug identifier, a numerical value for the given dose, and a flag which indicates whether the corresponding infusion ends here. Note that other relevant attributes, like the unit of the given dose and the administration route (intravenous, oral, etc.), are omitted here for the sake of simplicity

|  | case_id | infusion_id | timestamp | drug_id | dose | end |
| --- | --- | --- | --- | --- | --- | --- |
| 0 | 59036 | 4354701 | 2235-07-14 10:48:32 | 1001591 | 0.0 | N |
| 1 | 59036 | 4354701 | 2235-07-14 12:14:26 | 1001591 | 4.068 | N |
| 2 | 59036 | 4354701 | 2235-07-14 12:16:05 | 1001591 | 0.15 | Y |
| 3 | 59036 | 6300470 | 2235-07-14 12:16:07 | 1001725 | 100.0 | Y |
| 4 | 11067 | 6875044 | 2178-10-02 08:00:49 | 1002086 | 0.0 | N |
| 5 | 11067 | 6875044 | 2178-10-02 08:21:21 | 1002086 | 0.137 | N |
| 6 | 11067 | 6875044 | 2178-10-02 08:28:11 | 1002086 | 0.052 | N |
| 7 | 38354 | 5514092 | 2204-02-21 09:09:50 | 1001725 | 100.0 | Y |
| 8 | 38354 | 5514092 | 2204-02-21 09:09:50 | 1001980 | 5.78 | Y |

Drugs take effect either immediately or after a couple of minutes, depending on the administration route; furthermore, the effect duration also varies between drugs. From a machine learning point of view, the time when a drug has an effect is at least as important as when the drug is administered. We manually classified the 250 most frequently administered drugs into ‘short’ (10 minutes) and ‘long’ (24 hours) duration of effect, and specified intravenously administered drugs as taking effect immediately and all others as taking effect after 30 minutes. Based on this setup, the actual effect time can be determined for every administration from the raw medication data and added as a new feature. We also found that the number of different drugs is far too large for reasonable usage in training machine learning models, so for our specific use cases, we manually distributed them into five groups based on their effect on blood pressure (ranging from ‘strongly lowers blood pressure’ to ‘strongly raises blood pressure’). Other groupings might be necessary in other use cases.

### Diagnoses

Diagnoses are typically represented by ICD-10 codes [38], a hierarchical classification list with codes for all sorts of diseases. Indeed, in most diagnoses data we exported for our research, ICD-10 codes are available; in the remaining entries, however, only short free-text descriptions can be found. We, therefore, implemented a mechanism for automatically extracting ICD-10 codes from short German diagnosis texts, which proceeds by first ‘normalizing’ the text under consideration (convert to lower-case, remove special characters, replace frequently used synonyms) and then employing a predefined, automatically generated lookup table from normalized texts to ICD-10 codes to find the matching code. With this simple approach, roughly 15-25% of the missing codes can be imputed.

ICD-10 codes consist of a single letter from A to Z, followed by two to four digits. The letter and first two digits specify the disease group, whereas the remaining digits (if any) further specify the particular disease within its group. Due to the large number of different codes appearing in our raw data, we opted to consider only the disease groups represented by the first three characters of each code, decreasing the total number of diagnoses roughly by a factor of four. This way, we reduce the data complexity for subsequent machine learning and facilitate constructing the mapping mentioned above from free-texts to codes.

### Waveforms

Waveforms and vitals signs are exported as JSON files from the Bedmaster system and are converted into the by far more time- and memory-efficient HDF5 format. Waveforms are split into two-second ‘chunks’ in the raw data, which are timestamped lists of 2*fs* numerical values, *fs* being the sampling rate (e.g., 240 Hz). After creating the HDF5 files, consecutive chunks that are exactly two seconds apart are merged into contiguous numerical arrays. Temporal distances exceeding two seconds indicate interruptions in the waveform recording (e.g., because the bedside monitoring was turned off for a short period of time), and hence the corresponding chunks cannot be merged; see Supplementary Figure 1 (left) for a graphical depiction. However, we also observed that sometimes the timestamps of two consecutive chunks are either one or three seconds apart, leading to short overlaps and gaps. Visually inspecting these cases revealed a smooth transition of the signals from one chunk to the next. We, therefore, hypothesize that the timestamps are wrong and consequently merge chunks anyway. This is depicted in Supplementary Figure 1 (right).


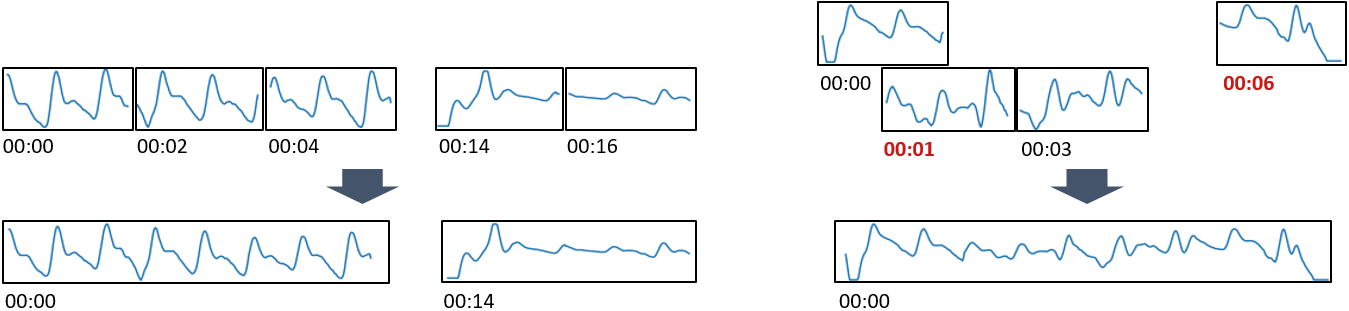


Supplementary Figure 1. Merging consecutive waveform chunks. Left: Consecutive chunks are merged if they are two seconds apart. They are not merged if they are farther apart. Right: One-second overlaps and gaps indicate wrong timestamps, so the corresponding chunks are merged anyway.

Finally, we stress that validating waveform signals is a complex task that cannot be achieved by simply checking whether individual values lie in a specific allowed range. Supplementary Figure 2, for example, shows an excerpt from a lead-I ECG signal with highly unusual curve morphology. Automatically detecting periods of such unusual waveform signals is challenging, especially since it must be done for signals of different kinds (e.g., ECG, blood pressure, SpO2, etc.). Although data-driven approaches to solving this problem exist [36], we have not systematically tried them on our data so far. Instead, we feed the raw, unprocessed signals to downstream supervised machine learning algorithms (e.g., LSTMs [39]) and rely on their ability to learn how to differentiate between normal and abnormal signals. Incorporating automatic detection mechanisms into data preprocessing is left for future work.


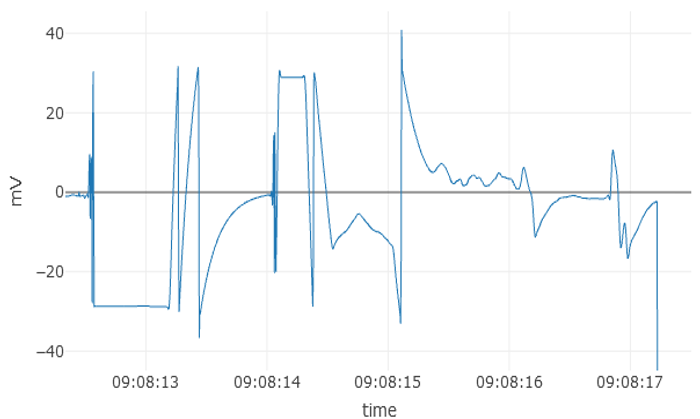


Supplementary Figure 2. Unusual lead-I ECG signal.
